# The impact of the Covid-19 pandemic on the uptake of routine maternal and infant vaccines globally: A systematic review

**DOI:** 10.1371/journal.pgph.0000628

**Published:** 2022-10-21

**Authors:** Amira Yunusa, Christie Cabral, Emma Anderson

**Affiliations:** 1 University of Bristol, Bristol, United Kingdom; 2 NIHR Health Protection Research Unit in Behavioural Science and Evaluation, University of Bristol, Bristol, United Kingdom; 3 Centre for Academic Child Health, University of Bristol, Bristol, United Kingdom; University of New South Wales, AUSTRALIA

## Abstract

Maintaining routine vaccination coverage is essential to avoid outbreaks of vaccine-preventable diseases. We aimed to understand the international impact of the COVID-19 pandemic on routine vaccination in pregnant women and children aged 0-5-years-old. A systematic review of quantitative and mixed methods studies exploring changes in vaccination coverage, vaccination services, and vaccine confidence since the start of the Covid-19 pandemic was conducted. MEDLINE, EMBASE, CINHAL, PsychINFO, Web of Science, Google Scholar, World Health Organisation, UK Government Joint Committee on Vaccination and Immunisation (including EU and US equivalents), and SAGE Journals were searched between 15-17th June 2021. Selected studies included pregnant women, health professionals, and/or infants aged 0-5-years-old including their parents (population); reported on the Covid-19 pandemic (exposure); presented comparisons with pre-COVID-19 pandemic period (comparator) and reported changes in routine maternal and infant vaccination coverage, services, and confidence (outcomes). Sources published only in non-English language were excluded. The Newcastle Ottawa Scale was used to assess study quality and risk of bias (ROB), and a narrative synthesis was undertaken. This review has been registered with PROSPERO (CRD42021262449). 30 studies were included in the review; data from 20 high-income countries (HICs), seven low- and middle-income countries (LMICs), and three regional studies (groups of countries). 18 studies had a low ROB, 12 had a higher risk, however both low and high ROB studies showed similar results. Two studies meeting the inclusion criteria discussed changes in routine vaccinations for pregnant women while 29 studies discussed infants. Both groups experienced declines in vaccination coverage (up to -79%) with larger disruptions in the accessibility and delivery of vaccination services reported within LMICs compared to HICs. Changes in vaccine confidence remained unclear. The COVID-19 pandemic resulted in decreased vaccine coverage and reduced routine vaccination services for pregnant women and infants, impacts on vaccine confidence requires more research.

## Introduction

Maternal, and infant vaccines have proven to be a powerful mechanism in decreasing infant morbidity and mortality [1, 2]. Routine vaccinations, as stated by the World Health Organisation (WHO), are ‘the sustainable, reliable, and timely interactions between the vaccine, those who deliver it and those who receive it to ensure every person is fully immunised against vaccine-preventable diseases [3]. The tetanus toxoid, reduced diphtheria toxoid, and acellular pertussis (Tdap) vaccination is an example of a routine vaccination administrated to expecting mothers, which is highly effective (91.4%; 95% confidence interval [CI] 19.5% to 99.1%) at preventing pertussis during an infant’s first two months of life, a disease capable of causing hospitalisation and death in this vulnerable population [1, 4]. Any decrease in vaccine coverage is a public health concern for increasing the risk of outbreaks of vaccine-preventable diseases, placing vulnerable individuals at further risk as they no longer benefit from herd immunity and contributing potential for extra strain on healthcare systems [2, 4].

The COVID-19 pandemic resulting from the novel severe acute respiratory syndrome coronavirus 2 (SARS-CoV-2) resulted in over 452 million confirmed cases globally and approximately 6 million reported deaths up to March 2022 [5]. With the widespread impacts of the pandemic, resources have been diverted from existing services, and concerns have been raised regarding the continuous coverage, service access, and delivery of routine vaccinations [2]. These concerns correspond with previous outbreaks, for example, in 2014 the Ebola virus disease epidemic in West Africa resulted in decreases in the delivery of maternal services, and vaccine administrations for diseases such as polio where reductions of -3,594 doses (-216 to -5,879 95% [CI], p = 0.0362) were reported in Guinea [6]. Lesson learnt from this epidemic included ensuring communication between service providers and these communities are maintained throughout outbreaks to maintain vaccination coverage [6]. Decreases in vaccination coverage were also reported in Sierra Leone during this outbreak period, for example a decrease in measles vaccine coverage from 71.3% (62.1% - 80.4% 95% [CI]) to 45.7% (29.2% - 62.2% 95% [CI]) [7]. Similar lessons to Guinea were learned with the addition of the necessity of higher quality supplementary immunisation activities, active surveillance to identify areas with low coverage, and the addition of a further dose for routine measles vaccine [7].

Prior to the pandemic, vaccination coverage rates were higher in high-income countries (HICs) than in lower-middle income countries (LMICs) [8, 9]. For example, diphtheria, pertussis, and tetanus third dose (DTP3) vaccine coverage in infants was 95%, in HICs and 73% in LMICs in 2017 [8]. Coverage sits even lower in low-income countries not receiving GAVI aid, with their combined DTP3 coverage in infants sitting at 48%, in 2017 [8]. Measles-containing-vaccine first dose (MCV1) coverage in infants within the African region was reported to be 74% compared to 95% in the European region in 2018 [9]. Therefore, vaccination coverage between HIC and LMIC regions was already inequitable. LMICs experience greater challenges with a lack of access to reliable transportation links, household crowding, and lack of economic means which contribute to existing inequalities in health and opportunities between these regions [10, 11]. These extra challenges mean loss of vaccination coverage in LMICs is *more* of a public health concern due to increased risks of disease exposure and lack of access to healthcare [10, 12].

This review takes a global approach to achieve a comprehensive overview of the impacts of the pandemic on routine vaccination and how impacts may differ between LMICs and HICs, where vaccination inequity was an existing issue. There is a historical lack of research on LMICs generally gives more reason to explore the global evidence [13]. With increasing globalisation, disease outbreaks in any area can affect the rest of the world, bringing responsibility for countries to work together in mitigating and controlling the impacts of pandemics and outbreaks to avoid global health issues [12, 14].

We need to understand changes in routine maternal and infant vaccinations since the COVID-19 pandemic to understand what is happening globally. It is important to evaluate the available evidence to highlight areas for improvement and targeting interventions. This can equip policy makers, health service commissioners, and the wider public health community to make informed decisions on the upkeep of these essential services and their accessibility throughout disease outbreaks.

This systematic review aimed to understand the impacts of the COVID-19 pandemic, specific to the SARS-CoV-2 species, on routine maternal and infant vaccination coverage, services, and confidence. We have defined vaccination coverage as changes in the proportion of vaccinated infants within their respective age group for their respective vaccination, vaccination services as any health service facilitating the administration of routine vaccines to infants, and vaccination confidence as changes in the attitude or behaviour of parents or healthcare workers surrounding the administration of infant vaccinations.

## Methods

Guidelines established by the Cochrane Handbook for Systematic Reviews of Interventions were used [15]. This review has been registered with PROSPERO (CRD42021262449).

### Selection criteria

The following inclusion criteria, based on the PICO (Population, Intervention/Exposure, Comparison, Outcomes) model were applied [16]:

- Population: pregnant women, health professionals, and infants aged 0-5-years-old, including their parents. This age range was chosen for the inclusion of many of the early routine vaccinations administered across vaccination schedules of most countries [17].
- Exposure: defined as the COVID-19 pandemic, as declared by the WHO on 11^th^ March 2020 [18].
- Comparison: defined as the pre-COVID-19 pandemic period, any period prior to March 2020 where the WHO declared a global pandemic, this has also been defined by the studies included themselves [19].
- Outcomes: changes in routine maternal and infant vaccination coverage, vaccination services (for example, operating hours, changes in delivery schedules), and/or vaccine confidence.

The WHO definition of routine vaccination (as stated above) was used [3]. Quantitative and mixed methods studies were included to gather all relevant quantitative results, and all countries were included for a global perspective. Studies were excluded if:

- They were not presented using English language to avoid translation error,
- They focused on other coronaviruses, for example SARS-CoV-1.
- The study PICO differed from those specified above.
- The sole focus was on non-routine vaccinations administration, considered as vaccinations not found on routine vaccination schedules such as post exposure prophylaxis, including the recent COVID-19 vaccine.

### Search strategy

A search strategy was created using relevant medical subject headings (MeSH) [20] and free text search terms, examples include pregnant, infant, vaccination, and COVID-19 (see S1 Text), to identify suitable studies. Databases and sources searched include OVID Medline (R and Epub Ahead of Print, In-Process, In-Data-Review and Other Non-Indexed Citations and Daily), EMBASE, CINHAL, PsychINFO, Web of Science (Social Science Citation Index), Google Scholar, WHO, UK Government JCVI (including EU and US equivalents), and SAGE Journals. Pre-prints were included within searches to gather all relevant data. Searches occurred between 15-17th June 2021, and publications up to the search date were included (excluding all studies published pre-2020 due to irrelevance to COVID-19). Retrieved studies were uploaded to the reference management tool EndNote. Duplicate studies were removed, and remaining studies were screened using their titles and abstracts to decide upon their relevancy to the review; this process was carried out only by AY due to resource constraints. Decisions were recorded using a PRISMA flow diagram [21]. Full texts of relevant studies were retrieved for full eligibility checks following title and abstract screening. Decisions around inclusion of studies where eligibility was less clear were made via team discussion (all authors) by strictly comparing these studies to our pre-defined PICO and considering resource constraints in the potential widening of this PICO for the inclusion of these studies.

### Data extraction

The author, year of publication, country, country income (based on World Bank 2021 classification) [22], study purpose, data collection methods and sources, population, sample size, exposure, control, outcomes, and other data of importance were extracted on to a data extraction form using Microsoft Excel by one researcher (MSc AY) due to resource constraints (see Table A in S1 Table). This enabled the comparison of differences between studies. Summary estimates, including confidence intervals, and p-values of quantitative studies were extracted where possible for the comparison of effect estimates between pre- and post-COVID-19 periods. Only quantitative data were extracted from mixed method studies.

### Quality assessment

A Risk of Bias (ROB) assessment, using the Newcastle-Ottawa Scale (NOS) [23], was applied to all included studies, as recommended by the Cochrane handbook [15]. The NOS adapted for cohort studies was applied to one study which specified itself as a cohort study. This was scored out of a maximum of 9 stars [23]. The NOS adapted for cross-sectional studies was applied to all other studies as they either defined themselves as cross-sectional or were not explicitly clear on their study type but could be identified as cross-sectional studies [24]. These studies were scored out of a maximum of 10 stars [24]. On the NOS scale, a score of ten stars represents low ROB while zero stars represents very high ROB [23]. The NOS simultaneously acted as a quality appraisal tool [23].

### Data synthesis

A narrative synthesis was conducted focusing on vaccine coverage, vaccination services, and vaccine confidence as outcomes. This was appropriate due to the variation between studies in their chosen methods of reporting (cumulative counts vs rates), while also allowing cohesive discussion of interactions between the different outcome measures stated [25, 26]. Tabulation of data was used throughout to assist with the presentation of results and to enable comparison between HICs and LMICs. Due to heterogeneity between studies and results, a meta-analysis could not be conducted [15]. The influence of ROB on the results of the review were explored.

## Results

**Fig 1:**
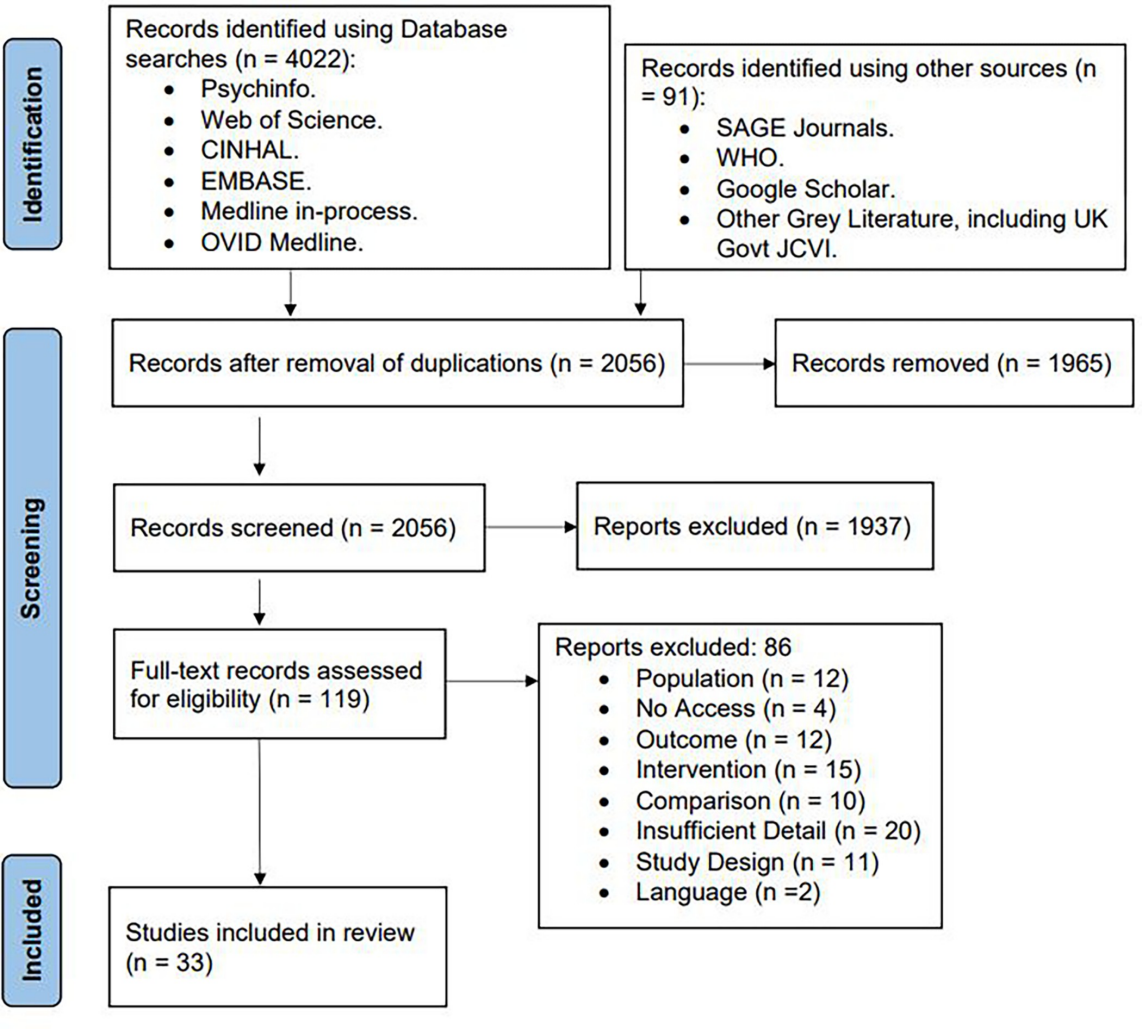
Preferred Reporting Items for Systematic Reviews and Meta-Analyses (PRISMA) flow diagram. This PRISMA flow diagram presents the study selection process [10].

4112 studies were retrieved: 4021 from database searches, 91 from other sources including governments and organisations (Fig 1). 2056 duplicates were removed, leaving 2056 studies for title and abstract screening where a further 1937 studies were removed. 119 studies underwent full text screening, excluding a further 89 studies after assessment. Reasons for exclusion after full text assessment include differing populations (for example pre- and late teens), outcomes measuring non-routine vaccinations, differing exposures such as the implementation of systems during the pandemic, and comparisons between post-COVID-19 periods, not pre-COVID-19 pandemic periods. Four studies were excluded due to no access for public use, 20 studies contained insufficient detail due to lacking quantitative results, 11 studies were purely qualitative, and two studies had language restrictions. 30 studies were ultimately included in the review [27–56].

### Study characteristics

Study characteristics are presented in Table 1 along with the main study results. Two studies were classified as descriptive analysis studies [32, 51], one as an interim analysis [35], eight as observational studies [27] (including ecological [37], cross-sectional [33, 39, 53, 54, 56], and cohort studies [31]), two as mixed method studies (one descriptive analysis [36], one cross-sectional [43]), and one as a retrospective review [48]. Sixteen studies did not specify their study type, and so were categorised as observational studies [28–30, 34, 38, 40–42, 44–47, 49, 50, 52, 55] (see Table 1). Twenty studies focused on HICs (Japan [27], US [28, 29, 32, 38, 40–42, 44], Netherlands [30], Singapore [31], Canada [33], England [34–36, 43], South Korea [37], Sweden [39], Italy [45, 46]), seven focused on LMICs (Pakistan [47, 50], South Africa [48], Brazil [49], Nigeria [51], Turkey [52], Lebanon [53]), and three studies focused on larger geographical areas including multiple countries which will be referred to as regional studies (global [56], Africa [55], South-East Asia and Western Pacific [54]), listed in Table 1. One study analysed multicentre data from a number of vaccination service providers (9 healthcare facilities across Singapore) [31], 19 studies analysed routinely collected data (eight at national-level [30, 34, 35, 37, 39, 41, 49, 55], and 11 at regional-level within the country [27–29, 32, 38, 40, 47, 48, 50–52]). Ten studies reported project-level data by independently gathering participants and collecting data through means such as surveys, questionnaires, and individual and service provider records [33, 36, 39, 42, 43, 45, 46, 53, 54, 56].

**Table 1:** Summary of studies included in the systematic review.

Study, Year (Reference)	Country or Region	Study Design	Study Population	Sample Size	Source and Method of Data Retrieval	Data Collection Period	Outcome Measure Effect Direction	Key Outcomes	NOS Score
						Pre-COVID-19 Period	Post-COVID-19 Period			Vaccine Coverage	Vaccine Services
**High-income Countries (HICs)**											
Aizawa et al., 2021 [27]	Japan, (Kawasaki, Niigata, Nagasaki, Fuchu regions).	Retrospective observational.	Children aged 0–15 years old.	Regional. 1,647,890 Children aged 0–15 years old. • Kawasaki (n = 101,083) • Niigata (n = 788,053) • Nagasaki (n = 462,071) • Fuchu (n = 296,683)	Regional, Japanese National Immunisation Programme; Kawasaki and Niigata health centres, the Nagasaki municipal office, and Fuchu City Medical Association records.	2016 to 2019.	January to September 2020.	N/A	↔	Changes in mean vaccines administered (%): Aged 3–4 years old, JE1-3: • ***Kawasaki*: *1%*** • Niigata: -0.5% • Nagasaki: -5.5% • Fuchu: -3.5% Aged 5–6 years old, MR2: • ***Kawasaki*: *5*.*5%*** • ***Niigata*: *3*.*5%*** • ***Nagasaki*: *2%*** • ***Fuchu*: *7%***	8
Bode et al., 2021 [28]	United States (Columbus, Ohio).	ANS; cross-sectional.	12 Clinical sites (>90,000 children and adolescents), focusing on 16 month olds data.	Regional.	Regional, Nationwide Children’s Hospital Paediatric Primary Care Network Records.	March 2017 to March 2020. (Includes post COVID-19 restrictions’ June to August 2020).	April to May 2020.	↓	N/A	Average proportion of 16-month-old children with MMR vaccination 72.0% (March 2017–20) vs 66.8% (April-May 2020) (p<0.001). Proportion with MMR vaccine by 16-months-old by insurance (p < 0.001): • Private; 74.4% (OR 1, reference) • Medicaid (Public); 71.2%, adjusted Odds Ratio of 0.79 (95% CI 0.60–1.04). • Self-pay: 66.6%; 0.59 (95% CI 0.44–0.78).	8
Langdon -Embry et al., 2020 [29]	United States (New York).	ANS; ecological.	1,600 Immunisation facilities, focusing on children aged 0–24 month olds data.	1,600 facilities.	Regional, Citywide Immunisation Registry.	2019.	2020.	N/A	↓	• 62% decrease in doses administered in 0-24-months-olds. • 46% decrease in facilities administering least one vaccine in 0-24-month-ods.	8
Middeldorp et al., 2021 [30]	Netherlands.	ANS; ecological.	Children aged under 2 years old.	National.	National, National Immunisation Register records.	January to July 2019.	March to September 2020.	↓	N/A	• 6% to -14% decrease in MMR1 vaccination uptake.	8
Zhong et al., 2021 [31]	Singapore.	Retrospective cohort.	Healthcare facilities with patients aged 1–2 years old.	9 Healthcare facilities; five public primary care clinics under the National University Polyclinics group, the paediatric outpatient clinic at National University Hospital, Singapore Hospitals, and three private paediatrician practices.	Multicentre, Healthcare facility records.	January to April 2017–2019.	January to April 2020.	N/A	↓	Number of MMR/MMRV given: Polyclinics: • 25.6% (95% CI -28.1% to -23.3%) decrease, April 2019 and April 2020. Hospitals: • 57.3% (95% CI -65% to -50%) decrease April 2019 to April 2020. Private clinics • 73.6% (95% CI -81% to -65.1%) decrease April 2019 to April 2020.	8
Nuzhath et al., 2021 [32]	United States (Texas).	Secondary data analysis.	Children aged 0–24 months.	1,369,881 total infants aged 0–24 months: Neonates (n = 325,922) 5-months (n = 342,906) 16-months (n = 364,611) 24 months (n = 336,442)	Regional, Texas Immunisation Registry (ImmTrac2, an opt-in register for healthcare providers, also contains data from Texas Department of State health Services, and Vital Statistical Unit) records.	May 2010.	May 2020.	↔	↓	• -5% decline in fully immunised 24-month-olds, ***2% increase in infants receiving birth dose of Hepatitis B***. • 27.2% (95% CI -24.3% to -30.2%) decline in doses administered to 5-month-olds; greater in rural counties (-28.6%; 95% CI −31.7% to −25.7%) than urban counties (−1.4%; 95% CI −1.7 to −1.2%). • 6.2% (95% CI -1.8% to -10.0%) decline in doses administered to 16-month-olds; greater in urban counties (-18.2%; 95% CI −19.1% to −17.3%) compared to rural counties (-12.1%; 95% CI −15.5% to −8.5%).	7
Piché-Renaud et al., 2021 [33]	Canada. (Ontario).	Descriptive, cross-sectional.	Family physicians and paediatrics.	457 responders.	Project-level, online survey.	N/A	May to July 2020.	N/A	↓	4% reported temporary closure of their practice. Of these, 61% stated this was due to a lack of PPE. 45% of respondents who offer vaccination to children mentioned a negative impact caused by the pandemic on the service. Of these, 5% closed their practice. 1% postponed all vaccines. 26% only provided vaccinations to children of a specific age, of these: 94% provided for 0–18 months old. 77% postponed vaccines for 4–6 year olds. Physicians from suburban practices were less likely to state a negative impact on their service in comparison to physicians in urban practices; adjusted odds ratio 0.61 (95% CI 0.39–0.97).	7
Public Health England, 2021 [34]	England.	ANS; retrospective observational.	Pregnant mothers.	National.	National, ImmForm using General Practices records.	2018 to 2019.	January to March 2021.	↓	N/A	Decline in monthly pertussis vaccination coverage (%) in pregnant women: • Jan 2019 vs 2021; -4.2% • Feb 2019 vs 2021; -6.2% • March 2019 vs 2021; -3.4%	7
Public Health England, 2021 [35]	England.	Interim analysis.	Children aged 0–24 months old.	National.	National, ImmForm and The Phoenix Partnership records.	2019.	2020 to 2021.	↓	N/A	87.3% completion of 3 doses of the Hexavalent vaccine by 6-months-old. -1.3% lower than 2020, -0.8% lower than 2019. 86.4% MMR1 vaccine coverage. -1.8% lower than 2020, -0.8% lower than 2019.	7
Skirrow et al., 2021 [36]	England (London).	Descriptive analysis, mixed methods.	London General Practices, focusing on children aged 0–5 years old data.	830 responders.	Project-level, online survey.	N/A	May 2020 (survey), August to November 2020 (interviews).	N/A	↓	6% reported issues with patients cancelling or not coming in. 5% of practices innovated their models of delivery; immunisations provided outside, drive-through or walk-through models, at different sites, or collaborations with primary care networks.	7
Yu et al., 2020 [37]	South Korea.	Ecological.	Children aged 0–6 years old.	National.	National, Korean Ministry of Public Administration and Security, and the Korean Disease Control and Prevention Agency records.	January to June 2019	January to June 2020	↔	N/A	6-months-old vaccine coverage (% change): • ***BCG*: *1*.*3% increase*.** • ***HepB (1st dose) 1*.*1% increase*.** • ***DTaP (1st dose) 1*.*1% increase*.** • ***Hib (1st dose) 1*.*1% increase*.** • ***PCV (1st) 1*.*2% increase*.** 12–15 months old vaccine coverage (% change): • MMR (1^st^ dose) -0.2% decrease. 12–23 months old vaccine coverage (% change): • HepA (1^st^ dose) -0.2% decrease. • JE (1^st^ dose) -0.3% decrease. 24–35 months old vaccine coverage (% change): • ***HepA (2nd dose) 1*.*4% increase*.** • ***JE (3rd dose) 0*.*3% increase*.** 48–72 months old vaccine coverage (% change) • DTaP (5^th^ dose) -1.9% decrease. • MMR (2^nd^ dose) -1.7% decrease. • JE (4^th^ dose) -1.4% decrease.	7
Bramer et al., 2020 [38]	United States (Michigan).	ANS; ecological.	Children aged 1–24 months.	Average sample size of 9,269 for the study period years 2016–2019, and 9,539 for 2020.	Regional, Michigan Care Improvement Registry records.	May 2016 to May 2019.	May 2020.	↔	N/A	% changes in infants vaccinated, 2019 vs 2020: ***1-month-olds*: *1%*.** 24 months-old: -8%. Up-to-date status for recommended vaccines declined -18.2% between 2019 to May 2020 for 5-month-olds. MCV coverage decreased -5.2% between 2019 to 2020 for 16-month-olds.	6
Falkenstein Hagander et al., 2021 [39]	Sweden.	Cross-sectional.	Regional Child Health Offices (staff including physicians, specialist nurses and psychologists).	National.	Project-level, web-based survey.	N/A	March to August 2020	↑	↓	***Increase in first dose coverage of MMR vaccine by 0*.*7% between June 2019 and June 2020*.**	6
Murthy et al., 2021 [40]	United States.	ANS; ecological.	Children aged 0–18 years old., focusing on children aged 0–6 years old.	Regional, 10 US States (Idaho, Iowa, Louisiana, Michigan, Minnesota, New York City, North Dakota, Oregon, Washington, Wisconsin)	Regional, Immunisation Information Systems records.	March to September 2018–2019	March to September 2020	N/A	↓	Median decline in DTaP doses administered by -15.7% for 0–24 month olds, -60.3% for 2–6 year olds in all jurisdictions. (March-May 2018 vs 2019).	6
Santoli et al., 2020 [41]	United States.	ANS; ecological.	Children aged 0–18 years old, focusing on children aged 0–24 months old.	National.	National, Vaccines for Children Program and Vaccine Safety Datalink (contains both publicly and privately insured patients) records.	January to April 2019	January to April 2020	N/A	↓	Cumulative change in all measles-containing doses -100,000 (Jan), -400,000 (April).	6
Sokol & Grummon, 2021 [42]	United States.	ANS; cross-sectional.	Parents with children aged 5 months—6 years old.	2164 responders.	Project-level, online survey.	2019–2020	May 2020 to 2021	N/A	N/A	Changes in child’s 2020–2021 influenza vaccination intentions due to COVID-19 (p <0.001): For parent’s whose child did not receive the 2019–2020 Influenza vaccine: 34% (95% CI 30%– 37%) less likely. 45% no change. 21% (95% CI 18%– 24%) more likely. For parent’s whose child did receive the 2019–2020 influenza vaccine: 24% (95% CI 22–27%) less likely. 37% no change. 38% (95% CI 36–41% more likely.	6
Bell et al., 2021 [43]	England.	Cross-sectional, mixed methods.	Parents and guardians with children aged 0–18 months old.	1,252 responders.	Project-level, online cross-sectional survey.	N/A	April to May 2020	N/A	↓	23.9% reported difficulties in organising or accessing vaccination appointments. 28.3% had their appointment cancelled.	5
Vogt et al., 2020 [44]	United States.	ANS; cross-sectional.	Paediatric healthcare practices with children aged 0–6 years old.	1,933 practices.	National (Federally purchased vaccines; accounts for 86% US paediatric practices), Vaccines for Children Program records.	N/A	May 2020	N/A	↓	89.8% of practices were open, 10.2% closed (9.8% temporarily closed, 0.5% permanently closed). 61.7% of open practices offered reduced office hours for in-person visits. This was 63.7% in urban areas and 55.4% in rural areas. Of those who continued to offer services, 89.2% offered to children aged <12 months, 81.4% to children aged 1–2 years. 44% for 3–6 years old.	5
Bechini et al., 2020 [45]	Italy (Tuscany).	ANS; cross-sectional.	Paediatricians.	233 responders.	Project-level, semi-structured online questionnaires.	N/A	March to May 2020	N/A	↓	93.3% continued to vaccinate, 7% suspended services. Of those who continued, 17.8% administrated only first scheduled doses, 82.2% administered all scheduled vaccine doses. 223 paediatricians had enforced preventive measures: • Hand-sanitizers in waiting rooms and common areas (98.2%). • Scheduling visits to limit crowding in waiting rooms (98.2%). • Environmental sanitation (92.4%). • Physical distancing measures in waiting rooms (87.4%).	4
Russo et al., 2021 [46]	Italy.	ANS; cross-sectional.	Families with children aged 0–11 years old.	1,474 responders.	Project-level, Italian Paediatric Society social media survey/questionnaire.	N/A	April to June 2020	↓	↓	42.5% responded their vaccination service postponed their appointment. 13.5% responded their vaccination service had closed. 44% were reluctant to leave home due to travel restrictions or lack of guidance.	3
**Low-Middle-income Countries (LMICs)**											
Chandir et al., 2020 [50]	Pakistan (Sindh).	ANS; ecological.	Vaccinators with children aged 0–23 months old.	2755 vaccinators (1518 public and 151 private immunisation clinics). >3.1 million children.	Regional, Government of Sindh’s Zindagi Mehfooz (Safe Life) Electronic Immunisation Registry records.	September 2019 to March (22^nd^) 2020	March (23^rd^) 2020 to May 2020	↓	↓	Decline in immunization visits: • Rural areas, -54.9%. • Union councils and slums -53.8%. • 28.8% decrease in daily average maternal tetanus toxoid vaccinations. The mean age of the BCG vaccination was lower during lockdown; 4.3 vs 6.3 weeks (95% CI 1.93–2.07, p ≤ 0.01). Daily average vaccinator attendance was 7.4% (95% CI: 5.29% - 9.51%, p < 0.0001) lower during the lockdown compared to baseline. • 79.3% decline in outreach services during lockdown (range -73.0% to -83.5%), -32.1% (range -20.9% to -40.6%) decrease in fixed sites. Within fixed-sites largest decline was in BCG (-40.6%), smallest decline in measles 1^st^ dose, -20.9%.	9
Jensen et al., 2020 [48]	South Africa. (KwaZulu-Natal Province).	Retrospective review.	Health facilities providing paediatric care.	589 fixed primary healthcare clinics (PHCs), 22 community health centres 39 district hospitals 13 regional hospitals 1 tertiary hospital, 1 national central hospital 16 specialised hospitals.	Regional District Health Information System records.	January 2018 to February 2020	April to June 2020	↓	↓	Fully immunised <1 year coverage %: 91.5% (95% CI 89.2% - 93.7%) Jan 2018 –Feb 2020, 84.5% March–June 2020 (p = 0.01). Changes in mean service delivery, measles 1^st^ dose coverage % by district: • eTheKwini (urban) (accounts for 30% of KwaZulu-Natal under 5 years population), -37%. • Zululand (rural) (10% KwaZulu-Natal under 5 years population), -10%.	9
Silveria et al., 2021 [49]	Brazil	ANS; cross-sectional.	Families with children aged under 3 years old.	National.	National, Information System of the National Immunization Program records, and the EPICOVID-19 Survey.	January to June 2017 -2019	January to June 2020. August 2020 (survey)	↓	N/A	Changes in vaccine coverage between 2019 to 2020 (approximately): • BCG -24% 0–3 year olds: 19% (95% CI 17 to 21.1%) missed their vaccination. 21% (95% CI 19% to 23.1%) according to vaccination cards. Poorest quintile, 22.5% (CI 19.3 to 26.2%, p = 0.03) missed their vaccine. Wealthiest quintile, 15% (CI 11.6 to 19.1%, p = 0.03) missed their vaccination.	9
Chandir et al., 2020 [47]	Pakistan (Karachi).	ANS; ecological.	Vaccinators with children aged 0–23 months.	790 vaccinators (242 public and 86 private immunisation clinics). 429,447 children.	Regional, Zindagi Mehfooz (Safe Life) Electronic Immunisation Registry.	September 2019 to March (22^nd^) 2020	March (23^rd^) to May 2020	↓	↓	Mean number of daily immunisation visits (for all antigens) decreased by -52.8%. 18% of immunisation centres were closed. • 88.6% reduction in immunisation doses given by outreach services and -38.7% reduction for fixed-centre services. Decrease in the mean proportion of vaccinators who attended work during the lockdown compared with baseline; 78.7% vs 91.6% person-days.	8
Olaniyan et al., 2021 [51]	Nigeria (Oyo States).	Descriptive secondary analysis.	Individuals aged 9 months to 44 years, focusing on 0–5 year olds.	1,727,159 (aged under 5 years old).	Regional, Monitoring and Evaluation Unit of Oyo State Primary Health Care Board records.	July 2019	August 2020	↓	↑	Average coverage rates (before vs after): BCG; -3.7% decline ***Average routine immunisation planned fixed sessions (before vs after)*: *3*.*1% increase*.** ***Average proportion of outreach sessions conducted*: *4*.*2% increase*.**	8
Kara et al., 2021 [52]	Turkey (Ankara).	ANS; cross-sectional.	Family practitioner, paediatricians, and paediatric infectious disease specialists providing services to children aged under 24 months old.	2,860 family practitioners, 1,908 paediatricians, and 70 paediatric infectious disease specialists.	Regional, survey using WhatsApp and practitioner education/training. Vaccine records from Ankara Provincial Health Directorate.	March to May 2019	March to May 2020	↓	↓	% Change in vaccination rate compared (March, April, May 2019 vs 2020): • ***6-in-1 1st dose (2-month-olds)*: *-0*.*5%*, *0%*, *1*.*2%***	5
Mansour et al., 2021 [53]	Lebanon.	Cross-sectional.	Private paediatricians.	345 responders.	Project-level, electronically completed cross-sectional survey. Ministry of Public Health Immunisation data records.	October 2019	April 2020	↓	↓	• 77.4% decrease in the utilization of routine immunization services. % Reduction in rates of vaccine utilisation in the private sector: • OPV: -57.5% • 20% decrease in the utilization of routine vaccination services in the public sector:	5
**Regional Studies (groups of countries)**											
Harris et al., 2021 [54]	South-East Asia and Western Pacific.	Cross-sectional.	Sanofi Pasteur country teams. Individuals aged 0 months–over 18 year olds, focusing on 0–6 year olds.	19 countries. 8 HICs: Australia, Brunei, Hong Kong, Japan, New Zealand, South Korea, Singapore, Taiwan. 11 LMICs: China, Indonesia, Malaysia, Thailand, Cambodia, India, Myanmar, Nepal, Pakistan, Philippines, Vietnam.	Project-level, structured questionnaires.	N/A	February to June 2020 (Questionnaire conducted)	↓	↓	Absolute percentage reduction in vaccine coverage rates, median % (IQR): • OPV (infancy): -79% (-42% to -79%). Disruptions to antigens (vaccines), median % (IQR): • 0–8 weeks old: 78% (0–90%). • 9 weeks—23 months old: 93% (80–100%). • 2–6 years old: 93% (61–100%). Disrupted antigens (vaccines) by sector: • Public sector 79% • Private sector 83%	9
Masresha et al., 2020 [55]	Africa.	ANS; retrospective observational.	African country immunisation programmes. Children receiving MCV, BCG, DTP vaccines (<2 years old).	National.	National, Routine Immunization Program records.	January to March 2018–2020	April to June 2020	↔	N/A	Changes in the monthly mean number vaccinated with DPT3 and MCV1 (January to March 2020 vs April to June 2020): Countries with low coverage prior to the pandemic (DTP3, MCV1): • ***Angola; -12%*, *6%*** • ***Chad 6%*, *13%*** • ***DR Congo 1%*, *2%*** • ***South Sudan -7%*, *9%*** • ***Guinea -52%*, *-53%*** Countries with high coverage prior to the pandemic: • Eritrea -9%, **2%** • Kenya -2%, **10%**	7
Saso et al., 2020 [56]	Global.	Cross-sectional.	Members of the International immunising Pregnant Women and Infant Network (IMPRINT).	48 responses representing 18 countries – 13 LMICs (36 participants), 5 HICs (12 participants).	Project-level, online questionnaire.	N/A	April 2020	N/A	↓	% Reporting issues with maternal vaccination delivery: 50% total of which 53% LMIC, 42% HIC. % Reporting issues with new-born vaccination delivery: 33% total of which 42% LMIC, 8% HIC.	4

Table 1 presents the study characteristics of studies included in the systematic review including the author, year of publication, country/region, study population (ages in brackets represent the study population extracted for the purpose of this systematic review), sample size, the source and method of data retrieval, the dates of comparisons, outcome measure effect directions (includes vaccine coverage and vaccine services), key outcomes identified, and the NOS result allocated to the study. The country income level was labelled according to the World Bank [22]. N/A–Not Applicable, ANS: Author Not Specified (followed by independent researcher classification). The outcome term ‘vaccine coverage’ differs between studies, including vaccine rate, uptake, mean number vaccinated. Outcomes relating to vaccine confidence have been reported under key outcomes; a positive vaccine confidence means there was an increased preference for routine vaccines, negative confidence supports a decreased preference. Positive outcome directions are highlighted in bold italics. Studies have been grouped based on the country income level and ordered by NOS score from highest score (10, indicating low ROB) to lowest score (0, indicating high ROB) within each group.

Effect direction key: upwards arrow ↑ shows a positive effect direction, downwards arrow ↓ shows a negative effect direction, opposing arrows ↔shows no change or conflicting findings

Two studies researched routine vaccinations for pregnant mothers [34, 50], and 29 for infants aged 0-5-years-old [27–33, 35–56]. Fifteen study populations also included those above 5-years-old: data were only extracted for vaccinations administered to those aged 0-5-years-old from these studies [27–29, 36, 37, 40–42, 44–46, 51, 53, 54, 56] (Table 1). Nine studies lacked a defined pre-COVID-19 period, however, were included as they reported data on the impacts of the pandemic and made it clear data comparisons were made to general pre-COVID-19 periods [33, 36, 39, 43–46, 54, 56].

Vaccinations in the studies include:

- Mumps, Measles, and Rubella (MMR)
- MMR and Varicella (MMRV)
- Measles containing vaccine (MCV)
- Diphtheria, Tetanus, and acellular Pertussis (DTaP)
- Diphtheria, Tetanus, Pertussis, Polio, *Haemophilus influenzae* type b (5-in-1)
- 6-in-1 (equivalent of the 5-in-1 and Hepatitis B)
- Pneumococcal conjugate (PCV)
- Bacillus Calmette-Guérin (BCG)
- Hepatitis B (HepB and 1^st^ dose HBV0)
- *Haemophilus influenzae* type b (Hib)
- Hepatitis A (Hep A)
- Influenza
- Polio (including Oral Polio vaccine OPV, Inactivated Polio Vaccine IPV)
- Japanese Encephalitis (JE)
- Varicella
- Rotavirus (Rota-1).

### Risk of Bias

18 studies achieved a NOS score of 7 stars or above, and therefore could be considered as good studies with a low ROB [27–37, 47–51, 54, 55] (see Tables B and C in S1 Table). 12 studies obtained a score less than 7 stars indicating increased ROB due to: no statistical tests, poor comparability by disregarding relevant confounders (including the age of infants at time of vaccination, or service type as public or private), or sampling concerns (small sample size, or convenience sampling) reducing the representativeness of the study population [38–46, 52, 53, 56]. Studies are presented in Table 1 and Tables A-C in S1 Table in descending order of ROB score.

## Main results

Overwhelmingly, there has been a decline in routine vaccination coverage and services internationally, with LMICs suffering more than HICs (see Table 1). Findings are described in more detail below. Studies with a higher ROB followed a similar trend to those with low ROB meaning there were no outstanding differences between their outcomes.

### Vaccine coverage

Results have shown an overwhelming decrease in vaccine coverage. 18 studies examined infant vaccination coverage, representing all seven LMICs [47–53], nine HICs [28, 30, 32, 34, 35, 37–39, 46] and two larger regional studies [54, 55]. 17 of these studies (13 low ROB [28, 30, 32, 34, 35, 37, 47–51, 54, 55], 4 high ROB [39, 46, 52, 53]), reported decreases in vaccination coverage including for varicella, JE, PCV, HepA, BCG, HepB/HBV0, DTaP, Polio/OPV/IPV, and MMR/MMRV/MCV, though four of these also reported some mixed results [32, 37, 38, 55]. One study (with a higher ROB) reported only an increase in vaccination uptake (first dose MMR, US) [39] (see Table 1). Between all studies included in this review decreases up to -79% were seen across all vaccinations [28, 30, 32, 34, 35, 37, 38, 46–55].

We compared our most robust studies (ROB score ≥ 7) that reported changes in vaccination coverage between HICs and LMICs. Five of the six robust HIC studies of vaccination coverage showed a decline ranging from -1.8% (MMR1 schedule completion in infants by 6 months old, national immunisation data, England [35]) to -14% (MMR1 in infants < 2 years, national immunisation data, Netherlands [30]), with others in between (e.g., -5% decrease in fully immunised 24-month-olds, regional immunisation data, Texas, US [32]). One study showed slight increases of 0.3–1.4% across specific infant vaccinations in South Korea [37]. Five of the most robust LMIC studies showed a decline ranging from -3.7% (BCG coverage in < 5-year-olds, regional data, Oyo State, Nigeria [51]) to -24% (BCG coverage, Brazil [49]) decrease, with others in between (e.g., -7% decrease in fully immunised infants <1-years-old, regional data, KwaZulu-Natal Province, South Africa [48]). The large African regional study reported mixed results across countries for DTP3 and MCV1 vaccination coverage, including some decreases (up to -52% for DTP3, Guinea) and some increases (up to 13% increase for MCV1, Chad) [55]. Increases were explained by the authors as resulting from a lack of strict, extended lock-down periods, and COVID-19 cases [55]. See Table 1 for more details.

Harris et al.’s (low ROB) large regional study included both HICs and LMICs and reported an overall decline for DTP, OPV, IPV, and Measles vaccine coverage rates within all ages; the greatest being in OPV with a median decrease of -79% (IQR -42% to -79%) administered during infancy in participants from 19 different countries across South-East Asia and the Western Pacific [54]. The smallest decrease was reported within school-entry aged children receiving measles vaccination with a median decrease of -9% (IQR -3% to -31%), from the same study [54].

The two studies exploring maternal vaccination coverage, both reported decreases. Chandir et al., reported a -28.8% average decrease in maternal tetanus toxoid vaccinations in (LMIC) Pakistan, while Public Health England reported a -4.2% decrease in monthly maternal pertussis vaccination coverage in (HIC) England [34, 50].

Studies with a high ROB (ROB score < 7), show more conflicting findings in coverage, though still mainly indicating a decline.

### Vaccine service changes

Post-pandemic results show a decrease in vaccine administration and disruptions to services in comparison to the pre-pandemic period, as reported in 21 studies: two regional studies [54, 56], six LMICs [47, 48, 50–53], and 13 HICs [27, 29, 31–33, 36, 39–41, 43–46] (Table 1). 11 low ROB [27, 29, 31–33, 36, 47, 48, 50, 51, 54] and 10 high ROB [39–41, 43–46, 52, 53, 56] studies exploring changes in vaccination services stated a negative effect direction, representing decreases in administrations and difficulties in vaccination delivery and access. Aizawa et al., reported conflicting results, due to differences in the administration of vaccines between age groups; increases in 5-6-year-olds receiving the MR2 vaccine [27]. Examples of these disruptions follow below including the extent of how these differ based on the characteristics of the vaccine services (e.g., public or private sector) or the infants (e.g., age).

Results showed decreases in vaccine administrations from the pre-pandemic period; for example, a -15% to -7.5% decrease in BCG administration in Japan [27]. Some changes to vaccine schedules were seen such as the mean age of BCG vaccination administration decreased from 6.3-weeks prior to the lockdown, to 4.3-weeks-old (95% CI 1.93 to 2.07, p < 0.01) in Sindh, Pakistan [50]. Some service providers only continued vaccinations for certain ages; for example, Vogt et al., found 81.4% of services in the US offered vaccinations to 1–2-year-olds, whereas only 44% continued for 3–6-year-olds [44]. Likewise, Piché-Renaud et al., identified 94% of services in Ontario, Canada continued vaccinations for 0-18-month-olds, while 77% postponed vaccinations for 4–6-year-olds [33]. Overall, declines in vaccine administrations were reported across both LMICs and HICs. These were more common within LMICs as in some cases vaccination administrations increased in HICs. For example, a 2% to 7% increase in measles and rubella 2^nd^ dose (MR2) vaccine administrations for infants aged 5–6-year-olds across within-country regions in Japan (Kawasaki, Niigata, Nagasaki, and Fuchu) [27].

Declines in administrations were greater within private sectors [28, 31, 47, 50, 53, 54]. In Singapore the number of MMR/MMRV vaccines administered differed between polyclinics -25.6% (95% CI -28.1% to -43.3%), hospitals -57.3% (95% CI -65% to -50%), and private clinics -73.6% (95% CI -81.0% to -65.1%) [31]. Harris et al., reported 79% of public sector antigens (vaccinations) were disrupted, 83% within the private sector within the South-East Asian and Western Pacific region [54]. Decreases in vaccine administrations between public and private sectors were primarily seen within HICs, whereas in LMICs these differences in vaccine administrations by setting were typically reported between fixed and outreach services. Declines in administrations were greater for outreach services (-79.3% to -88.6% decrease) than for fixed-centre services in Pakistan (-32.1% to -38.7%) [47, 50]. Interestingly Olaniyan et al. reported a 3.1% increase in fixed and 4.2% increase in outreach vaccination *services* within Oyo State Nigeria, however, a decrease in vaccination *coverage* (-3.5%) for HBV0, could still be seen in this study [51].

Locations of vaccination services impacted the extent of service delivery in both LMICs [48, 50] and HICs [32, 44]. In Texas US, vaccine administration for 5-month-olds declined by -28.6% (95% CI -21.7% to -25.7%) in rural areas compared to -1.4% (95% CI -1.7% to -1.2%) in urban areas, while for 16-month-olds vaccination declined by -12.1% (95% CI -15.5 to -8.5) in rural areas, compared to -18.2% (95% CI -19.1% to -17.3%) in urban areas [32]. eTheKwini, an urban area in South Africa, reported a -37% decline in measles 1st dose coverage, whereas rural Zululand experienced a -10% decline [48].

Results show reductions in operating hours and increased duration of consultations [33, 36, 39, 43, 44, 46, 50, 51, 54, 56]. For example, Vogt et al., found across the US, 61.7% of practices offered reduced office hours for in-person visitations; of these, 63.7% were in urban areas, and 55.4% in rural areas [44]. Across other studies, Russo et al., found up to 42.5% of vaccination appointments were postponed or cancelled by vaccination services from their 1,474 survey responders in Italy, 13.5% stated vaccination services closed, while 44% of parents were reluctant to travel due to travel restrictions [46]. A lack of guidance was identified by England as Bell et al.’s online survey which found 25.6% of parents were unaware that childhood vaccinations continued throughout the pandemic [43]. From this same study, 23.9% to 53.3% of parents experienced difficulties accessing and booking their child’s vaccination appointment [43]. Logistical disruptions included: staff shortages, for example as identified by Saso et al. in their globally distributed questionnaire; equipment shortages, including personal protective equipment (PPE), and issues with the vaccine supply-chain [33, 36, 39, 54, 56]. Sindh, Pakistan experienced a -7.4% (95% CI -5.29% to -9.51%, p < 0.0001) decrease in the daily average vaccinator attendance, a common occurrence in LMICs [50].

### Vaccine confidence

Six studies addressed differences in vaccine confidence between the pre- and post-pandemic period; terminology included ‘vaccination intentions’, ‘importance of vaccination’, and ‘parent concerns’ [33, 39, 42, 43, 45, 52].

Some differences in vaccine confidence were reported among parents. Sokol and Grummon found 60% of parents intended to change their paediatric influenza vaccination behaviour due to the pandemic [42]. For parents whose children did not receive the 2019–2020 influenza vaccine 34% (95% CI 30%– 27%) responded that the pandemic made them *less* likely to have their child vaccinated for the 2020–2021 influenza vaccine compared with their plans before the pandemic, while 21% (95% CI 18% - 24%) responded they would be *more* likely [42]. Among parents whose children received the 2019–2020 influenza vaccine, 24% (95% CI 22% - 27%, p < 0.001) reported being *less* likely, while 38% (95% CI 35% - 41%) reported being *more* likely to have their children vaccinated with the 2020–2021 influenza vaccine [42].

Between practitioners, in Turkey, when asked ‘Which was the attitude of your patients regarding routine vaccination during the pandemic?’ 38.3% of family practitioners, 74.4% of paediatricians, and 65.8% of paediatric infectious disease specialists stated patients did not want to come in for vaccination due to the pandemic [52]. However, 57%, 10.5%, and 11.4% respectively also stated no problems with parental attitudes during the pandemic [52]. In Sweden, physicians reported parental concerns over their infant’s vaccination administration by comparing the post-pandemic attitude of parents to the pre-pandemic period, on a scale of 1 (not at all concerned) to 10 (very much concerned); 5% reported a score of 5, 10% a score of 4, 15% a score of 3, 40% a score of 2, and 30% a score of 1, signifying a fair proportion of parents with increasing concerns surrounding the vaccination of their infant following the pandemic [39].

## Discussion

At the time of development, this review was amongst the first of which we were aware to systematically explore the impacts of the COVID-19 pandemic on routine maternal and infant vaccination coverage, services and attitudes globally, serving as a rapid overview.

Our results from early data show that since the pandemic hit, routine maternal and infant vaccination coverage has decreased for all vaccinations in all settings investigated. The pandemic negatively impacted vaccination services, indicating problems with access and delivery. Both HICs and LMICs experienced decreases in vaccination coverage and difficulties with vaccine services. In some LMICS and HICS settings these changes were similar, however due to pre-existing low vaccine coverage in LMICs, lower coverage rates post-pandemic was reported within these settings in comparison to HICs. This is an important concern as the threshold for vaccination coverage must remain high for herd immunity to take place, additionally, it continues to highlight the poor access to healthcare and existing health disparities in vaccination coverage between these settings increasing global inequalities. Maintaining vaccination coverage in LMICs is thus even more important, though these are the countries suffering more declines.

Our findings suggest that private or self-funded services experienced larger declines in vaccine delivery compared to those receiving publicly funded healthcare, however it is advised more research is conducted in this area as in some countries, such as the UK, it was found that dependency on private or self-funded services increased due to difficulty in accessing public healthcare services, and longer waiting times due to the pandemic [57]. Outreach services were disproportionately affected compared to fixed-services typically due to unavailable staff; a common issue, particularly in LMICs. These results from our review are important as it may indicate those self-funding their child’s vaccinations in different countries may be less inclined to seek out routine vaccinations for their infants during the pandemic. This may be due to wider determinants such as financial insecurities resulting from the pandemic [58]. Additionally, the location of vaccination services played an unclear part in vaccine service accessibility; rural areas sometimes reported higher vaccination administrations in comparison to urban areas, with the opposite seen in other studies. The impact of the rural or urban location of vaccination services in this case is not clear indicating more research needs to be done in this area, for example, in the UK this could be achieved by reviewing changes in vaccination coverage for General Practices across the country between the pre-pandemic and post-pandemic periods; however, this may not be applicable for countries struggling to routinely collect this data. Previous research has shown that childhood vaccination coverage in LMICs has typically been lower in rural areas in comparison to urban areas, for example in the Western Pacific Region this can differ from around an average of 60% in rural areas to 70% in urban areas [59]. Interventions and policies in LMICs should therefore target those reliant on outreach services, while for both HICs and LMICs it should be ensured those on private or self-funded healthcare can access services during times of uncertainty to maintain coverage.

Reduced service operating hours and increased duration of consultations indicated were among the changes seen in vaccination services, resulting in fewer infants and pregnant women accessing routine vaccinations. Logistical issues including a lack of PPE, and disruptions to the vaccine-supply chain also contributed to lower vaccination uptake. Although countries continued with their vaccination schedules, not all parents were aware, indicating the importance of clear public health messages and the efficient allocation of resources.

The few studies reporting increases in vaccination coverage detected these in younger infants, where minimal increases (0.7% increase for 1^st^ dose MMR) [39] were reported in contrast to the larger magnitude of reported decreases seen in older infants receiving later doses (e.g., 79% decrease for OPV) [54]. This could be explained by the increased healthcare contacts in early life through mandatory routine development visitations which were utilised by health services as an opportunity for the administration of early routine childhood vaccinations, for instance as seen in the UK [60]. This finding highlights the importance of also working to maintain vaccine coverage in older infants in crises, although results showed some countries were also able to maintain vaccination coverage through the pandemic [36].

Results for changes in vaccine confidence between the pre and post pandemic period remain unclear due to a lack of available research; results simultaneously described both increases and decrease in vaccine confidence resulting from the pandemic. Even with inconclusive results, the majority of studies exploring changes in vaccine confidence were conducted within HICs so there is more of a research gap for LMICs.

Existing inequities between HIC and LIC regions have been exacerbated further by the COVID-19 pandemic [61]. We have gathered data on the impacts of the COVID-19 pandemic on routine maternal and infant vaccinations globally, however, further research is still necessary. This review found only six LMIC studies, compared to thirteen HIC studies, explored changes in vaccine services, highlighting the need for more evidence from these settings.

This is particularly the case for LMICs where more evidence describing changes in vaccine confidence, and accessibility to vaccination services is needed for a comprehensive understanding of the impacts. The data we have collated mirrors the magnitude of the impact of the pandemic on these maternal and infant services, however, these results are representative of many potentially unreported consequences of the pandemic. Our results align with Evans and Jombart’s recent modelling of expected versus actual global immunisation for DTP1, DTP3 and MCV1 in 2020, which indicated a global decline of 2.9% attributable to the pandemic with disproportionate impacts between LMICs (-3.8%, 95% [CI] 2.6% - 5.1%), and HICs (-0.9%, 95% [CI] -2.2% - 0.3%) [61].

International organisations such as the WHO have attempted to address the impacts of the pandemic on vaccination coverage by raising the importance of surveillance and by tailoring responses and plans in addressing vaccination gaps [9, 62–64]. The World Health Assembly has endorsed the ‘Immunization Agenda 2030’ for strategically addressing vaccine accessibility globally for 2021–2030 [62]. This makes recommendations of how to overcome challenges posed by infectious diseases outbreaks by setting country-specific targets for immunisations, ensuring efforts are people-focussed, driven by data, and partnership-based for sustainable coverage [62]. For example, ensuring health workforce availability, and strengthening leaderships and communication for immunisation services; two issues raised in this systematic review [62]. The measles outbreaks strategic response plan 2021–2023 acts as an exemplar, highlighting issues raised in the accessibility of vaccinations during the COVID-19 pandemic similar to those mentioned above and found throughout the results of this review [65]. The report provides a set of measurable objectives countries can work towards to improve the resilience of their vaccination services and responses to vaccine preventable diseases through improving access to funding, training tools, routine risk assessments, catch-up schedules for missed doses, and periods of intense routine immunisations when coverage levels are lower than target [65].

This systematic review has found that vaccination services for many countries were not prepared to withstand the impacts of a pandemic as declines in vaccination coverage and negative impacts on vaccination services were still reported across all countries included in this review [12]. The pandemic resulted in negative impacts on vaccination coverage, and vaccination services and inequalities between LMICs and HICs and global efforts need to address this. More research exploring the impacts on the pandemic on vaccine confidence is needed for the success of these efforts to ensure efforts are ‘people-focussed’ as mentioned in the Immunization Agenda 2030, to identify priorities in maintaining vaccine coverage and services throughout similar crises [62]. It may be beneficial for countries to focus on country-level analysis to identify those within the population experiencing the greatest inequalities in accessing these services, as well as to identify any disproportionate impacts on service providers within countries as trends may differ between countries.

Understanding how different regions have managed, and the consequence on routine vaccinations is important to inform health protection teams and policy makers, to better evaluate protocols and to adjust responses accordingly to minimise health impacts on routine vaccinations caused by pandemics and similar emergencies.

### Strengths and limitations

A strength of this review is the comprehensive investigation into an important health area impacted by the pandemic with potentially significant public health consequences. By conducting a quality assessment and comparing the outcomes of high and low ROB studies we were able to strengthen our conclusions.

Limitations include that due to resource constraints, one researcher conducted screenings and data extraction. While the methods would be strengthened by independent screening and data extraction by another researcher, cases of uncertainty were discussed in depth with two experienced researchers (co-authors EA and CC) to minimise this limitation. Due to time constraints, qualitative studies were not explored, which we recognise as beneficial to include in future research to provide richer detail on these findings. We identified a lack of research on maternal vaccinations so could not draw strong conclusions about the pandemic effects, though the existing research indicates cause for concern. We recommend more research be done in this area with the inclusion of qualitative studies for a richer explanation of results. Four studies identified during the literature search were not published for public use, while two studies were not presented using English language, resulting in potential missing evidence. Heterogeneity between studies prohibited the modelling of a comprehensive meta-analysis. In the future a review investigating the impacts of the pandemic between specific time periods, for example pre-lockdown vs lock-down periods, may assist in understanding the extent of the impacts of the pandemic.

## Conclusion

The COVID-19 pandemic has negatively impacted routine maternal and infant vaccination coverage and vaccination service globally. In LMICs where vaccine coverage was already lower than HICs, the impacts of the pandemic has been even more pronounced, increasing the likelihood of vaccine preventable disease outbreaks and increasing existing inequity. All countries will need to strategically collaborate for the better prevention and control of infectious diseases to avoid further epidemics and pandemics, but HICs will also have an ethical duty to assist LMICs in decreasing these widening global health inequalities. Implementing catch-up sessions in all settings to maintain vaccine coverage is imperative to protecting vulnerable populations and avert further health crises. Evidence found in this review expresses emergency response plans to situations such as that seen with the COVID-19 pandemic will need reviewing in all settings to minimise negative changes in infant vaccinations coverage and administration, and to protect against associated negative health outcomes.

## Supporting information

S1 Checklist The table used as a guide for conducting the research article. (DOCX)

S1 Text Search strategy. The search strategy conducted on the databases Medline, Embase, and PsychINFO (Medical subject headings (Mesh), text word (tw)).
(DOCX)

S1 Table Table A in S1 Table. Data extraction form. Table A provides a template of the data extraction form utilised using the software Microsoft Excel. As shown the following details were extracted: record number (relating to EndNote referencing), author, year of publication, country, country income level, methodology, study purpose, data collection methods and source, population, sample size, exposures, controls, outcomes (changes in vaccine coverage, services, and confidence), additional comments for data of significance, and the Newcastle-Ottawa Scale (NOS) Risk of Bias (ROB) score allocated to the study. Table B in S1 Table. NOS adapted for cohort studies result [23]. Table B shows the ROB assessment results for the Zhong et al., using the NOS adapted for Cohort studies. The maximum number of stars which can be retrieved is 9 indicating low ROB, 0 would be the minimum indicated high ROB. * Means star awarded,—means information unavailable. Table C in S1 Table. NOS adapted for cross-sectional studies results [23, 24]. Table C shows the ROB assessment for the 29 studies assessed using the NOS adapted for cross-sectional studies arranged from studies retrieving the greatest NOS score (10) to the lowest (0). * Means star awarded,—means information unavailable.
(DOCX)
