# Supplementary material for: The impact of the Covid-19 pandemic on the uptake of routine maternal and infant vaccines globally: A systematic review
Source: PLOS Glob Public Health. 2022 Oct 21;2(10):e0000628. doi: 10.1371/journal.pgph.0000628 (PMC10022285; doi:10.1371/journal.pgph.0000628)
Supplement: S1 Text — The search strategy conducted on the databases Medline, Embase, and PsychINFO (Medical subject headings (Mesh), text word (tw)). (DOCX) [file pgph.0000628.s002.docx]

### **S1 Text. Search Strategy**

The following is the search strategy conducted on the databases Medline, Embase, and PsychINFO. (Medical subject headings (Mesh), text word (tw)) [20]:

(Vaccines[Mesh] OR Vaccination[Mesh] OR vaccine uptake[Mesh]) AND (antenatal[tw] OR maternal[tw] OR pregnan*[tw] OR child*[tw] OR pre-school[tw] OR infant*[tw] OR 5 years old[tw] OR newborn[tw] OR bab*[tw] OR mother*[tw] OR parent*[tw] OR carer*[tw] OR guardian*[tw]) AND (COVID-19[Mesh] OR COVID-19[tw] OR coronavirus[tw] OR pandemic[tw] OR outbreak[tw] OR epidemic[tw] OR SARS-CoV-2[tw] OR coronavirus disease[tw]) AND (healthcare disparities[tw] OR vaccin*[tw] OR vacci* uptake[tw] OR vacci* access[tw] OR vacci* delivery[tw] OR immuni#ation[tw] OR immuni#ation progra*[tw] OR routine vacci*[tw] OR Mass Vaccination[Mesh] OR vacci* schedule[tw] OR vacci* coverage[tw] OR vacci* service*[tw] OR health service*[tw] OR health clinic*[tw]) AND (whooping cough[tw] OR pertussis[tw] OR influenza[tw] OR MenB[tw] OR meningitis[tw] OR rotavirus[tw] OR vaccine preventable disease*[tw] OR diphtheria[tw] OR hepatitis B[tw] OR hib[tw] OR haemophilus influenzae type b[tw] OR polio*[tw] OR tetanus[tw]) AND (attitude*[tw] OR opinion*[tw] OR perspective[tw] OR thoughts[tw]) limit to yr="2019 -Current".

Google Scholar search strategy: COVID-19 pandemic uptake routine maternal pregnant infant vaccines.

CINAHL search strategy: Covid-19 AND pandemic AND vaccines OR maternal immunizations AND global health AND infant AND attitudes or beliefs or perceptions.

Web of Science, Social Science Citation Index (SSCI) search strategy, (Title (TI), abstract (AB):

(AB=(antenatal OR maternal OR pregnan* OR child* OR pre-school OR infant* OR 5 years old OR newborn OR bab* OR mother* OR parent* OR carer* OR guardian* AND COVID-19 OR coronavirus OR pandemic OR outbreak OR epidemic OR SARS-CoV-2 OR coronavirus disease AND healthcare disparities OR vaccin* OR vacci* uptake OR vacci* access OR vacci* delivery OR immunisation OR immunisation progra* OR routine vacci* OR Mass Vaccination OR vacci* schedule OR vacci* coverage OR vacci* service* OR health service* OR health clinic* AND whooping cough OR pertussis OR influenza OR MenB OR meningitis OR rotavirus OR vaccine preventable disease OR diphtheria OR hepatitis B OR hib OR haemophilus influenzae type b OR polio OR tetanus AND COVID-19 OR coronavirus OR pandemic OR outbreak OR epidemic OR SARS-CoV-2 OR coronavirus disease AND attitude* OR opinion* OR perspective OR thoughts) AND (TI=(COVID-19 OR coronavirus OR pandemic OR outbreak OR epidemic OR SARS-CoV-2 OR coronavirus disease) AND (AB=(routine vaccine OR routine vaccination AND maternal OR infant OR pre-school OR aged 2-5 years) AND LANGUAGE: (English), Timespan=2019-2021
