# Supplementary material for: The impact of the Covid-19 pandemic on the uptake of routine maternal and infant vaccines globally: A systematic review
Source: PLOS Glob Public Health. 2022 Oct 21;2(10):e0000628. doi: 10.1371/journal.pgph.0000628 (PMC10022285; doi:10.1371/journal.pgph.0000628)
Supplement: S1 Table — Data extraction form. Table A provides a template of the data extraction form utilised using the software Microsoft Excel. As shown the following details were extracted: record number (relating to EndNote referencing), author, year of publication, country, country income level, methodology, study purpose, data collection methods and source, population, sample size, exposures, controls, outcomes (changes in vaccine coverage, services, and confidence), additional comments for data of significance, and the Newcastle-Ottawa Scale (NOS) Risk of Bias (ROB) score allocated to the study. Table B in S1 Table. NOS adapted for cohort studies result [23]. Table B shows the ROB assessment results for the Zhong et al., using the NOS adapted for Cohort studies. The maximum number of stars which can be retrieved is 9 indicating low ROB, 0 would be the minimum indicated high ROB. * Means star awarded,—means information unavailable. Table C in S1 Table. NOS adapted for cross-sectional studies results [23, 24]. Table C shows the ROB assessment for the 29 studies assessed using the NOS adapted for cross-sectional studies arranged from studies retrieving the greatest NOS score (10) to the lowest (0). * Means star awarded,—means information unavailable. (DOCX) [file pgph.0000628.s003.docx]

**Table A in S1 Table. Data extraction form.** Table A provides a template of the data extraction form utilised using the software Microsoft Excel. As shown the following details were extracted: record number (relating to EndNote referencing), author, year of publication, country, country income level, methodology, study purpose, data collection methods and source, population, sample size, exposures, controls, outcomes (changes in vaccine coverage, services, and confidence), additional comments for data of significance, and the Newcastle-Ottawa Scale (NOS) Risk of Bias (ROB) score allocated to the study.

| **Record Number.** | **Author.** | **Year.** | **Country.** | **Country Income Level.** | **Study Design (Methodology).** | **Study Purpose.** | **Data Collection Methods and Source.** | **Population.** | **Sample Size.** | **Exposure.** | **Control.** | **Results for Changes in Vaccines Coverage.** | **Results for Changes in Vaccine Services.** | **Results for Changes in Vaccine Confidence.** | **Further Comments.** | **Risk of Bias** |
| --- | --- | --- | --- | --- | --- | --- | --- | --- | --- | --- | --- | --- | --- | --- | --- | --- |
|  |  |  |  |  |  |  |  |  |  |  |  |  |  |  |  |  |
|  |  |  |  |  |  |  |  |  |  |  |  |  |  |  |  |  |
| **Table A in S1 Table. Data extraction form.** Table A provides a template of the data extraction form utilised using the software Microsoft Excel. As shown the following details were extracted: record number (relating to EndNote referencing), author, year of publication, country, country income level, methodology, study purpose, data collection methods and source, population, sample size, exposures, controls, outcomes (changes in vaccine coverage, services, and confidence), additional comments for data of significance, and the Newcastle-Ottawa Scale (NOS) Risk of Bias (ROB) score allocated to the study. | | | | | | | | | | | | | | | | |

**Table B in S1 Table. NOS adapted for cohort studies result [23].** Table B shows the ROB assessment results for the Zhong et al., using the NOS adapted for Cohort studies. The maximum number of stars which can be retrieved is 9 indicating low ROB, 0 would be the minimum indicated high ROB. * Means star awarded, - means information unavailable.

| **Study** | **Selection**  **(Maximum 4 stars)** | | | | **Comparability**  **((Maximum 2 stars)** | **Outcome**  **(Maximum 3 stars)** | | | **Summary**  **(Out of 9 stars)** |
| --- | --- | --- | --- | --- | --- | --- | --- | --- | --- |
| **Author, Year.**  **(Reference)** | **Representativeness of exposed cohort.** | **Selection of the non-exposed cohort.** | **Ascertainment of exposure.** | **Demonstration that outcome of interest was not present at start of study.** | **Comparability of cohorts based on study design or analysis.** | **Assessment of outcome.** | **Was follow up long enough for outcomes to occur.** | **Adequacy of follow up of cohorts.** | **Total Stars.** |
| Zhong et al., 2021 [31] | - | * | * | * | ** | * | * | * | 8 |
| **Table B in S1 Table: NOS adapted for cohort studies result** [23]**.** Table B shows the ROB assessment results for the Zhong et al., using the NOS adapted for Cohort studies. The maximum number of stars which can be retrieved is 9 indicating low ROB, 0 would be the minimum indicated high ROB. * Means star awarded, - means information unavailable. | | | | | | | | | |

**Table C in S1 Table. NOS adapted for cross-sectional studies results [23,24].** Table C shows the ROB assessment for the 29 studies assessed using the NOS adapted for cross-sectional studies arranged from studies retrieving the greatest NOS score (10) to the lowest (0). * Means star awarded, - means information unavailable.

| **Study** | **Selection**  **(Maximum 5 stars)** | | | | **Comparability**  **(Maximum 2 stars)** | **Outcome**  **(Maximum 3 stars)** | | **Summary**  **(Out of 10 stars)** |
| --- | --- | --- | --- | --- | --- | --- | --- | --- |
| **Author, Year.**  **(Reference)** | **Representativeness of the sample.** | **Sample Size.** | **Non-responders.** | **Ascertainment of the exposure.**  **(Maximum 2 stars)** | **Comparability of studies.** | **Assessment of outcome.**  **(Maximum 2 stars)** | **Statistical Test** | **Total Stars.** |
| Silveira et al., 2021 [49] | * | * | * | ** | * | ** | * | 9 |
| Jensen et al., 2020 [48] | * | * | * | ** | * | ** | * | 9 |
| Harris et al., 2021 [54] | * | * | * | ** | ** | ** | - | 9 |
| Chandir et al., 2020 [50] | * | * | - | ** | ** | ** | * | 9 |
| Olaniyan Akintunde et al., 2021 [51] | * | * | * | ** | * | ** | - | 8 |
| Middeldorp et al., 2021 [30] | * | * | * | ** | * | ** | - | 8 |
| Bode et al., 2021 [28] | * | - | - | ** | ** | ** | * | 8 |
| Aizawa et al., 2021 [27] | * | * | * | ** | * | ** | - | 8 |
| Langson-Embry et al., 2020 [29] | * | * | * | ** | * | ** | - | 8 |
| Chandir et al., 2020 [47] | * | * | - | ** | ** | ** | - | 8 |
| Masresha et at., 2020 [55] | * | * | - | ** | * | ** | - | 7 |
| Yu et al., 2020 [37] | * | * | * | * | * | ** | - | 7 |
| Skirrow et al., 2021 [36] | * | - | * | ** | ** | * | - | 7 |
| Piché-Renaud et al., 2021 [33] | - | - | * | * | ** | ** | * | 7 |
| Nuzhath et al., 2021 [32] | * | * | - | ** | * | ** | - | 7 |
| Public Health England, 2021 [34] | * | * | - | ** | * | ** | - | 7 |
| Public Health England, 2021 [35] | * | * | - | ** | * | ** | - | 7 |
| Sokol & Grummon, 2020 [42] | - | * | * | - | ** | * | * | 6 |
| Santoli et al., 2020 [41] | * | - | - | ** | * | ** | - | 6 |
| Murthy et al., 2021 [40] | - | * | - | ** | * | ** | - | 6 |
| Bramer et al., 2020 [38] | * | - | - | ** | * | ** | - | 6 |
| Falkenstein Hagander et al., 2021 [39] | * | * | * | * | * | * | - | 6 |
| Vogt et al., 2020 [44] | * | - | * | * | * | * | - | 5 |
| Kara et al., 2021 [52] | - | - | - | ** | * | ** | - | 5 |
| Bell et al., 2021 [43] | * | - | - | * | * | * | * | 5 |
| Mansour et al., 2021 [53] | * | - | - | * | * | ** | - | 5 |
| Bechini et al., 2020 [45] | * | - | - | * | * | * | - | 4 |
| Saso et al., 2020 [56] | - | - | - | * | ** | * | - | 4 |
| Russo et al., 2021 [46] | * | - | - | * | - | * | - | 3 |
| **Table C in S1 Table:** **NOS adapted for cross-sectional studies results** [23,24]**.** Table C shows the ROB assessment for the 29 studies assessed using the NOS adapted for cross-sectional studies arranged from studies retrieving the greatest NOS score (10) to the lowest (0). * Means star awarded, - means information unavailable. | | | | | | | | |
